# Botulinum Toxin Treatment of Stiff Person Syndrome—A Critical Review and Update

**DOI:** 10.3390/toxins18030130

**Published:** 2026-03-05

**Authors:** Ava Grace Tohidian, Samira Marie Comtesse, Shahroo Etemadmoghadam, Bahman Jabbari

**Affiliations:** 1 School of Medicine, Royal College of Surgeons, D02 YN77 Dublin, Ireland; avagtohidian@gmail.com; 2 Faculty of Medicine, Goethe University, 60590 Frankfurt am Main, Germany; samira.comtesse@aol.com; 3 Glenn Biggs Institute for Alzheimer’s and Neurodegenerative Diseases, University of Texas Health San Antonio, San Antonio, TX 78229, USA; shahrooetemad@yahoo.com; 4 Department of Neurology, Yale University School of Medicine, New Haven, CT 06510, USA

**Keywords:** botulinum toxin, botulinum neurotoxin, stiff person syndrome, onabotulinumtoxinA, incobotulinumtoxinA, abobotulinumtoxinA, physiotherapy

## Abstract

Stiff person syndrome (SPS) is an autoimmune disorder with muscle stiffness and spasms, for which current therapies provide incomplete relief. Botulinum neurotoxin (BoNT) has been explored as an adjunctive symptomatic treatment. The aim of this review was to critically evaluate the clinical evidence for BoNT therapy in SPS. Using Medline, Scopus and Google Scholar, we identified nine reports that were published up to 1 January 2026. English articles and articles with information on study type, type/dose of BoNT and treatment results were included. One study was double-blind and placebo-controlled, one was retrospective and seven were single-case reports, comprising 46 patients. Open-label trials used botulinumtoxin-A (Botox, Dysport or Xeomin), while the blind study applied abobotulinumA (Dysport). All but one study (a case report) demonstrated motor improvement and a reduction in painful spasms associated with patient satisfaction. Reported doses ranged from 300 to 800 units for onabotulinumtoxinA and incobotulinumtoxinA and from 700 to 1000 units for abobotulinumtoxinA. The literature highlights the need for randomized clinical trials in larger cohorts, with careful selection of dose, injection sites, and adjunct physiotherapy, as well as an evaluation of early BoNT therapy in SPS. The novelty of this review lies in its critical synthesis of reported data and inclusion of most recent reports.

## 1. Introduction

**Figure 1:**
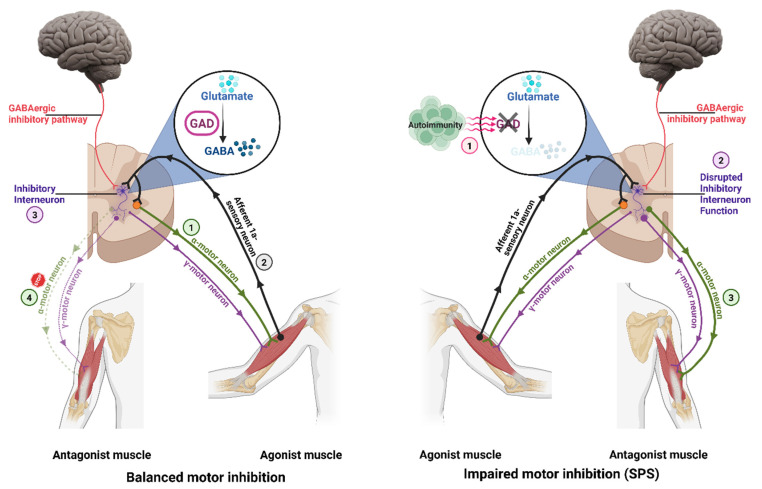
The left panel illustrates physiological conditions in which GABA synthesized from glutamate by glutamic acid decarboxylase (GAD) inhibits antagonistic muscles during motor activity. The right panel depicts stiff person syndrome (SPS) in which diminished GABAergic signaling fails to exert this inhibitory effect and leads to increased activity of alpha motor neurons. Created in BioRender. Etemad, S. (2026) https://BioRender.com/anc8yhu (accessed on 3 January 2026).

Stiff person syndrome (SPS) is a rare autoimmune disorder characterized by impaired inhibitory neurotransmission in the central nervous system, primarily due to antibodies targeting glutamic acid decarboxylase (GAD), which is the enzyme responsible for synthesizing gamma-aminobutyric acid (GABA) [1,2,3]. Paucity of GABA in the spinal cord leads to a lack of inhibition of antagonist muscles (through inhibitory interneurons) during a motor act, leading to progressive increase in the muscle tone (Figure 1).

Under the umbrella term of stiff person spectrum disorders (SPSD), classic SPS represents a heterogeneous condition that is associated with a wide range of clinical manifestations. Among SPSD phenotypes, classic SPS is not only the most frequent but often the most challenging form, as it typically has an insidious onset with complex symptoms [1]. The reduction in GABAergic inhibition leads to the hyperexcitability of motor neurons, manifesting clinically as progressive muscle stiffness, and stimulus-sensitive painful spasms [4]. High-titer anti-GAD antibodies are found in most patients, and these antibodies are thought to interfere with GABA synthesis, resulting in decreased GABA levels in the brain and cerebrospinal fluid [5].

Due to the absence of standardized diagnostic criteria, SPS frequently remains under- or misdiagnosed, thereby limiting robust population-based epidemiological studies [6]. Nevertheless, SPS is known to be very uncommon, with an estimated prevalence of roughly one–two cases per million and a female predominance of approximately 2:1 [7]. Onset typically occurs in mid-adulthood, around the age of 40 years [8]. SPS does not predominantly occur in any racial or ethnic group [6]. Diagnostic delay frequently results in irreversible disability, making early diagnosis and the prompt implementation of an appropriate therapeutic regimen all the more important [1].

Treatment of SPSD is multimodal and typically combines GABAergic symptomatic therapies, immune-based treatments and non-pharmacological interventions, tailored to the individual disease burden and phenotype. Benzodiazepines (particularly diazepam) remain the cornerstone of symptomatic management, while targeted physical and rehabilitative strategies (e.g., stretching, gait and balance training, heat and aqua therapy, deep tissue techniques and related modalities) are important adjuncts [9]. In patients with insufficient control under symptomatic therapies, escalation to immunomodulatory approaches should be considered. These most commonly include intravenous or subcutaneous immunoglobulins, plasma exchange, corticosteroids and B-cell-directed or other steroid-sparing agents such as rituximab, mycophenolate or azathioprine. Stem cell transplantation is generally reserved for highly refractory cases. Emerging longitudinal data suggest that SPSD may be a progressive disorder and that an earlier initiation of immune therapies could mitigate disability progression, although this requires confirmation in further studies [10]. In addition to systemic, symptomatic and immunomodulatory therapies, localized interventions, such as botulinum toxin (BoNT) injections, have been explored to address focal stiffness and painful spasms in SPS [11]. Considering the clinical and immunological heterogeneity of stiff person spectrum disorders, response to BoNT may vary across disease subtypes, antibody profiles, and patterns of muscle involvement, and its role in SPS remains incompletely defined.

Given the limited and heterogeneous literature on botulinum neurotoxin therapy in stiff person syndrome, the aim of this review is to provide an updated and critical synthesis of published reports evaluating botulinum neurotoxin therapy in stiff person syndrome spectrum disorders, including recent larger case series. We focus on treatment efficacy, injection strategies, dosing paradigms, adverse effects, and methodological limitations, and outline the priorities for future clinical investigation.

## 2. Research Design

We searched Medline, Scopus and Google Scholar for papers published up to 1 January 2026. The search terms consisted of botulinum toxin, botulinum neurotoxin and stiff person syndrome. Two of the authors (AT and BJ) independently searched the literature. Two other authors (SC and SE) verified the search results. Excluded from the search were articles in any language other than English and review manuscripts. The technical issues related to botulinum toxin therapy in SPS, as well as the strengths and weaknesses of the reported studies, are provided in the Section 4 of this manuscript. The novelty of this review is to provide the latest data in the literature including the only report that includes a sizeable number of patients.

## 3. Results

We found nine manuscripts that conformed to the search criteria. These articles were published between 1993 and 2025. A summary of the search findings is displayed in Table 1 which includes the authors’ names, the date of publication, the number of studied patients, the type of study, the type of toxin used, the toxin dose, the injected muscles and outcome measures, the results of botulinum toxin therapy in SPS, and any adverse effects (AEs) of the treatment. The retrieved data consisted of one double-blind, placebo-controlled study, one retrospective study and seven single-case reports [Table 1].

**Figure 2:**
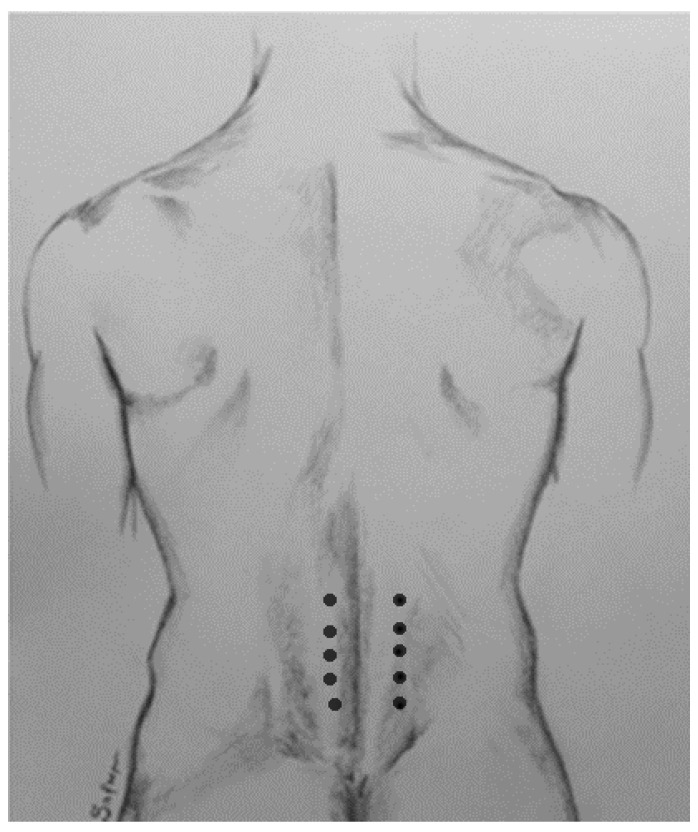
The sites of low back injections into the lumbar erector spinae muscles [11]. Drawing courtesy of Damoun Safarpour, M.D.

In 1993, Davis and Jabbari [11] first reported a significant reduction in muscle tone and painful muscle spasms and an improvement of gait in a 36-year-old male with SPS following injection of onabotulinumtoxin-A into the lumbar paraspinal (erector spinae-ES) muscles. The patient’s serum anti-GAD antibody levels (assessed at Mayo Clinic) were 1/122,000 (normal < 1/120). The total injected dose of the toxin was 560 units over three weeks (160, 200, 200 units per week). In each session, ES muscles were injected at all five lumbar levels with one fifth of the dose injected per level (Figure 2). A repeat injection with 200 units (100 units/side) 6 months later again reduced the tone of the lumbar ES muscles and frequency of painful muscle spasms and improved gait.

In 1997, Liguori et al. [12] studied the effect of an intramuscular injection of abobotulinumtoxinA in two patients with SPS under a double-blind, placebo-controlled protocol. Both patients had increased anti-GAD antibodies. One of the patients had severe stiffness in both lower limbs, whereas the second demonstrated severe stiffness in the muscles of both upper limbs associated with frequent painful spasms in the affected muscles. The patients’ muscle tone was assessed by Unified Parkinson Disease Rating Scale (UPDRS) and the frequency of their muscle spasms by a spasm frequency scale (0–5) in which five represented 30 or more spasms/day. The patients received botulinum toxin injections into the muscles on one side, whereas the muscles on the other side received saline. In the third post-injection day, patients reported a marked reduction in painful muscle spasms on the side of the toxin injections, a finding that was associated with a notable reduction in muscle tone in the involved muscles (rectus femoris, biceps femoris and adductors for the lower limbs). In the second week, the muscles on the saline-injected side also showed reduced tone and spasms, although not as much as the toxin-injected side, a finding that the authors attributed to the spread of the toxin to the spinal cord. The total toxin dose/session was 700 and 1000 units for the patient with upper and lower limb involvement, respectively. Patients reported no adverse effects.

Since 2013, six other reports described improvement of SPS symptoms after an intramuscular injection of BoNTs, each in a single patient [Table 1] [13,14,15,16,17,18].

Recently, Roman et al. [19] reported the results of a retrospective study in 37 patients with SPS, 83.8% of whom had the classic variant. The patients received either onabotulinumtoxinA or incobotulinumA into the paraspinal muscles, hip flexor muscles and shoulder girdle muscles. The patients’ response to treatment was assessed by Patient Global Impression of Change (PGIC) and a 5-point Likert scale in which five represented 76–100% satisfaction. Repeat injections were performed every 2.5–3 months up to approximately two years (eight treatment sessions). Nine patients dropped out after the first treatment, five of them cited the cost of the treatment and the distance to the treatment facility as the cause. Of those who continued treatment, the rate of significant satisfaction (Likert four or five) was 67.9% after the first treatment, 95% after the third treatment and 100% after the eighth treatment. Investigators escalated the toxin dose during returned visits, as the mean total dose was 400 units for the first injection, 600 units for the third injection and 800 units for the eighth injection. The authors concluded that a higher injection dose correlated with better outcomes. BoNT injections did not influence the level of anti-GAD antibodies. None of the patients developed clinical resistance. Adverse effects occurred in eight patients after the first injection and four patients after the third injection, respectively but none after the eighth injection. They mostly included local pain and spasms that abated after a few days. One patient reported transient mild leg weakness, and one experienced a transient flu-like syndrome.

## 4. Discussion

The increased muscle tone and muscle stiffness in SPS are related to the failure of the GABA system to relax antagonist muscles; hence, it is different from the rigidity and spasticity seen in extrapyramidal and upper motor neuron disorders. Currently, the leading disease-modifying treatments for SPS include IVIG infusions and a treatment with monoclonal antibodies. The former, which has been in use now for over 30 years, has shown to decrease muscle tone and painful spasms as well as improve the quality of life in many patients affected by SPS [20,21]. Careful observations of sizable cohorts with SPS have shown, however, that approximately one third of the patients do not respond to IVIG treatment, and in a quarter of responders, IVIG treatment over months or years loses its effectiveness [22]. Among monoclonal antibodies, Rituximab has shown promise in open label studies [23] but failed to show significant improvement in a carefully designed blind investigation [24]. Symptomatic treatment of muscle stiffness and muscle spasms with benzodiazepines (for example, diazepam) is helpful, but in many patients, optimum response requires using large doses for which most patients have poor tolerance.

In 1949, Burgen et al. [25] first showed that the paralytic effect of botulinum neurotoxins is due to blocking the release of acetylcholine at the neuromuscular junction [25]. Over the past 30 years, several phase III studies in a large number of patients have shown that BoNT treatment of spastic muscles can improve the quality of life in adults and children with spasticity regardless of the etiology [26,27,28]. Furthermore, long-term studies with repeated injections over months and years have demonstrated the sustained effect of BoNT therapy in post-stroke spasticity [29,30]. Although the mechanism of increased tone in spasticity is different from the stiffness seen in SPS (failure of GABA), it is reasonable to predict that BoNT therapy, by reducing muscle tone, can improve the condition of patients with SPS. In fact, eight out of nine studies in this review have presented data to support this notion (Table 1). Moreover, the efficacy of an intramuscular injection of onabotulinumtoxinA in reducing associated painful muscle spasms has been shown in other conditions such as diabetic neuropathy [31]. Although the botulinum toxin does not address the underlying central loss of inhibitory control in SPS, targeted reduction in peripheral muscle overactivity may attenuate reflex amplification and co-contraction, thereby providing focal symptomatic benefit.

Considering the paucity of the literature in BoNT therapy for SPS, there is a need for conducting well-designed, double-blind clinical trials to discern the role of BoNT therapy in SPS. Since the success of BoNT therapy weighs heavily on the selection of the right muscles and the application of the right dose, before commencing such studies, the investigators need to define the best muscles for injection and ascertain the optimum dose that is likely to produce satisfactory results. Furthermore, as emphasized by Dalakas [32], investigators need to use a Validated Stiffness Index in order to make results more reliable.

The data from the studies of sizeable number of patients with SPS indicates that paraspinal muscles are always involved in SPS [33]. Unlike the involved limb muscles, where affected muscles are usually easy to discern, recognition of culprit paraspinal muscles in SPS may prove challenging and require careful electromyographic screening. Furthermore, insufficient dosing needs to be avoided as it can lead, erroneously, to study failure. Along the same line, although for treatment of spasticity FDA recommends a limit of 400 units for onabotulinumtoxinA and incobotulinumtoxinA per session (1500 units for abobotulinumtoxinA), studies of patients with severe spasticity have shown that up to 800 units per session of incobotulinumtoxinA can be safely used [34]. One of the studies in our review [19] (Table 1) also used doses of up to 800 units of onaA or incobotulinumtoxin A in SPS and reported no adverse effects [Table 1]. Although the doses of different BoNTs are not truly interchangeable [35], in most clinical trials a dose approximation of 1:1 between the two above-mentioned toxins (ono and incobotulinum toxin) has been used in several clinical trials.

A point of concern is that repeated injections of high doses of BoNTs over time (if such doses are needed for optimum response in SPS) may produce neutralizing antibodies and lead to unresponsiveness. However, in a study of more than 5000 patients receiving repeated injections of the new batch of onabotulinumtoxinA (introduced to the market in 1997), with doses up to 600 units, the incidence of neutralizing antibody formation was 0.3%, and just 0.1% of the studied patients demonstrated clinical unresponsiveness [36]. This concern may not even be an issue with incobotulinumtoxinA, which is practically devoid of neutralizing antibodies [37].

Since the mechanism of progressive stiffness in SPS is failure of the GABA system, any chronic treatment for SPS should not further impair spinal GABA transmission. In the spinal cord, GABA is made from the excitatory neurotransmitter glutamate via the function of glutamic acid decarboxylase (GAD), a function that requires sufficient vitamin B6. It has been shown that peripherally injected BoNTs (A or B) reach the spinal cord via retrograde transmission, but the presence of the cleaved toxin in the spinal cord does not affect glutamate release in the motor part of the cord [38]. Furthermore, there is no evidence that the presence of BoNT in the spinal cord directly affects the function of GABAergic neurons [39].

The importance of physiotherapy as an adjunct to BoNT treatment has been shown in many studies of post-stroke spasticity [40,41,42]. Unfortunately, with the exception of a single-case report [17], physiotherapy has not been mentioned in any other studies cited in this review (Table 1). Future studies of BoNT therapy in SPS should include physiotherapy and preferably the use of modern physiotherapeutic techniques [43]. Furthermore, electromyography is a powerful tool to locate hyperactive muscles, and ultrasound is a useful instrument to confirm muscle anatomy. Only four of nine studies included in this review mentioned EMG in their protocol. Future studies should use EMG and ultrasound to enhance the accuracy of the injection sites.

The weakness of this review does not include the non-English literature or the fact that most reviewed articles are single-case reports or open label trials, hence leading to biased conclusions. The strength of the review is the critical assessment of the published literature and the inclusion of the latest information on BoNT treatment of SPS.

## 5. Conclusions

The data from limited open label studies and a small, blind study suggests that different type-A botulinum toxins can improve disturbing symptoms of SPS, such as painful muscle spasms and impaired gait. These promising results need to be validated by randomized clinical trials using standardized measures of stiffness in the research protocol. Inclusion of physiotherapy in the treatment plan is likely to enhance the effect of BONT therapy in SPS.

## Figures and Tables

**Table 1:** Botulinum toxin Treatment of stiff person syndrome.

Authors and Year	Type of Study	Pts	Toxin	Total Dose in Units	Muscles Injected	Mode of Assessment	Results
Davis and Jabbari, 1993 [11]	OL	1	onaA	560 injected in three sessions: (160, 200 and 200) into 5 sites	Lumbar erector spinae muscles	Intensity of exertional back pain and frequency of spontaneous muscle spasms. Intensity of EMG discharges	Near complete cessation of exertional back pain and spontaneous painful spasms lasting about 4 months; similar results were seen in the 2 subsequent injections, 4 months apart.
Liguori et al., 1997 [12]	DB-PC	2	aboA versus saline which was injected into contra-lateral stiff muscles	Patient 1: 700 Patient 2: 1000	Patient 1: Right adductor magnus, adductor longus, rectus femoris, vastus lateralis, vastus medialis, long and short heads of biceps femoris, tibialis posterior, soleus, and gastrocnemius Patient 2: Right trapezius, deltoid, biceps, coracobrachialis, brachioradialis	Muscle tone: Unified Parkinson’s Disease Rating Scale (UPDRS). Severity of spasms using spasm frequency scale	Both patients showed a significant reduction in muscle tone and painful spasms following aboA injections into the affected muscles. In the second weak post injection, saline-injected muscles also showed fewer spasms and a reduction in tone. Repeat injections resulted in the same improvements.
Anagnostou et al., 2012 [13]	OL	1	aboA	1st: 400 2nd: 550 3rd: 900	Vastus lateralis, vastus medialis, and rectus femoris	Patient subjective reporting, Modified Ashworth Scale, EMG	1st, injection: no improvement; 2nd injection (4 months later): caused moderate improvement in rigidity; 3rd injection: resulted in drastic improvement in rigidity, gait, and painful spasms.
Pakeerappa et al., 2015 [14]	OL	1	onaA	300 U (every 3 months)	Masseter and paraspinal muscles	Patient subjective reporting of the severity of stiffness and pain	Stiffness and painful spasms improved and continued to improve with repeated injections every 3 months.
Shah et al., 2016 [15]	OL	1	onaA	NM	Long head of biceps femoris, adductor magnus, and rectus femoris	Severity of stiffness	Increased ROM, significant pain relief, and decreased stiffness
Esplin et al., 2017 [16]	OL	1	incoA	300 U	Left biceps, left brachioradialis, flexor digitorum superficialis and profundus	Joint range-of-motion (ROM) and patient subjective reporting of degree of stiffness and pain	Injections cause only mild improvement of symptoms.
Marvulli et al., 2024 [17]	OL	1	aboA	240 U	Cervical and thoracic paravertebral muscles	Numeric Rating Scale (NRS), joint range-of-motion (ROM), Modified Ashworth Scale (MAS), and BB dynamic stiffness mensuration	Significant improvement of pain, spasms, and joint mobilization for 6 months post-injection. At 8 months post-injection, the effects of the aboA treatment wore off, and symptoms returned
Nguyen et al., 2025 [18]	OL	1	onaA	250–300	Biceps femoris, soleus, lumbar and dorsal paraspinals	Patient reported level of stiffness and quantitative ADLs	Significant improvement of stiffness and ADL after each 3-month injection
Roman et al., 2025 [19]	OL	37	OnaA and incoA	visit 1: 200 u visit 3: 380 u visit 8: 400-80	Varied: Paraspinal, hip flexors and shoulder girdle muscles	Patient-reported outcome approach converted into 5-point Likert scale	94% of patients who attended 3+ visits had a positive response, and 100% of patients who attended 8+ visits had a positive response

OL: open label; DB-PC: double-blind, placebo-controlled; onaA: onabotulinumtoxinA (Botox); incoA: incobotulinumtoxinA (Xeomin); aboA: abobotulinumtoxinA (dysport); ADL: activities of daily living; and ROM: range of motion.

## Data Availability

The original contributions presented in this study are included in the article. Further inquiries can be directed to the corresponding author.
